# Immunization Status of Children Admitted to a Tertiary-care Hospital of North India: Reasons for Partial Immunization or Non-immunization

**DOI:** 10.3329/jhpn.v28i3.5560

**Published:** 2010-06

**Authors:** Devendra Kumar, Anju Aggarwal, Sunil Gomber

**Affiliations:** Department of Pediatrics, University College of Medical Science and Guru Teg Bahadur Hospital, Delhi 110 095, India

**Keywords:** Child, Immunization, Vaccination, India

## Abstract

Reasons for the low coverage of immunization vary from logistic ones to those dependent on human behaviour. The study was planned to find out: (a) the immunization status of children admitted to a paediatric ward of tertiary-care hospital in Delhi, India and (b) reasons for partial immunization and non-immunization. Parents of 325 consecutively-admitted children aged 12–60 months were interviewed using a semi-structured questionnaire. A child who had missed any of the vaccines given under the national immunization programme till one year of age was classified as partially-immunized while those who had not received any vaccine up to 12 months of age or received only pulse polio vaccine were classified as non-immunized. Reasons for partial/non-immunization were recorded using open-ended questions. Of the 325 children (148 males, 177 females), 58 (17.84%) were completely immunized, 156 (48%) were partially immunized, and 111 (34.15%) were non-immunized. Mothers were the primary respondents in 84% of the cases. The immunization card was available with 31.3% of the patients. All 214 partially- or completely-immunized children received BCG, 207 received OPV/DPT1, 182 received OPV/DPT2, 180 received OPV/DPT3, and 115 received measles vaccines. Most (96%) received pulse polio immunization, including 98 of the 111 non-immunized children. The immunization status varied significantly (p<0.05) with sex, education of parents, urban/rural background, route and place of delivery. On logistic regression, place of delivery [odds ratio (OR): 2.3, 95% confidence interval (CI) 1.3–4.1], maternal education (OR=6.94, 95% CI 3.1–15.1), and religion (OR=1.75, 95% CI 1.2–3.1) were significant (p<0.05). The most common reasons for partial or non-immunization were: inadequate knowledge about immunization or subsequent dose (n=140, 52.4%); belief that vaccine has side-effects (n=77, 28.8%); lack of faith in immunization (n=58, 21.7%); or oral polio vaccine is the only vaccine required (n=56, 20.9%. Most (82.5%) children admitted to a tertiary-care hospital were partially immunized or non-immunized. The immunization status needs to be improved by education, increasing awareness, and counselling of parents and caregivers regarding immunizations and associated misconceptions as observed in the study.

## INTRODUCTION

Immunization has been one of the most significant and cost-effective public-health interventions to decrease childhood morbidity and mortality. Approximately three million children die each year of vaccine-preventable diseases. Recent estimates suggest that approximately 34 million children are not completely immunized, with almost 98% of them residing in developing countries (1). The World Health Organization (WHO) launched the Expanded Programme on Immunization (EPI) in 1974 with focus on the prevention of six vaccine-preventable diseases of the childhood by 2000. This was implemented by the Government of India in 1978 (2). On 19 November 1985, the Universal Immunization Programme was introduced in India, aiming at covering at least 85% of all infants by 1990. Further, a national sociodemographic goal was set up in the National Population Policy 2000 to achieve universal immunization of children against all vaccine-preventable diseases of the childhood by 2010 (3).

The coverage of vaccination in India is far from complete despite the commitment for universal coverage. According to the National Family Health Survey (NFHS) 3, only 43.5% of children, aged 12–23 months, were fully vaccinated—57.5% in urban areas and 38.6% in rural areas (4). Reasons for lack of coverage vary from logistic ones to those dependent on human behaviour. A number of previous studies have explored the reasons for non-immunization (5–9) but none has been carried out on children admitted to a tertiary-care hospital. Hence, the present study was undertaken to assess the status of immunization and to analyze the various factors responsible for the suboptimal coverage of immunization among admitted patients.

## MATERIALS AND METHODS

### Study setting and sample

This four-month study was conducted from April to July 2007 at a tertiary-care hospital (University College of Medical Sciences and Guru Tegh Bahadur Hospital, New Delhi, India). Of 1,266 child patients admitted to the paediatrics ward for various ailments, 325 consecutive patients, aged 12–60 months, were selected for the study. Demographic and socioeconomic data were recorded using a questionnaire. The immunization status of the enrolled patients was assessed as per the national immunization programme (2). Mother was the primary respondent; if the mother was not available, father was interviewed informally by the author (DK) after the acute phase of illness in the child was over. Mothers were asked about the immunizations received by their children by one year of age, and where possible, this information was verified by cross-checking against the vaccination cards of the children. Children who had received BCG and three doses of DPT/oral polio vaccine (OPV) and measles vaccine as scheduled in the first year of life were classified as fully immunized. Those who had missed any dose of six primary vaccines were labelled as partially immunized, and those who had not received any vaccine, except OPV in pulse polio immunization, up to 12 months of age, were defined as non-immunized (8). If the child was partially immunized or non-immunized, the reasons for the same were recorded using open-ended questions.

### Analysis of data

For a power of 80% and alpha of 0.05, sample size was calculated as 288 using a previous study (9) in which the proportion of completely-immunized children was 25%. Statistical analysis was carried out using the SPSS software (version 13). The p value of <0.05 was considered significant. Chi-square test and logistic regression analysis were done to determine the statistical significance of the association between the immunization status and the recorded demographic details.

### Ethics

The study was approved by the ethical committee of the Guru Tegh Bahadur Hospital, New Delhi. Written informed consent was taken from the parents of the study subjects.

## RESULTS

Of the 325 consecutively-admitted children, aged 12–60 months, 148 (45.54%) were male and 177 (54.46%) were female. The mean±standard deviation of age was 34.14±12.96 months. Mothers were the primary respondents in 273 (84%) cases and fathers in 52 (16%) cases. Only 104 (32%) cases were from Delhi, and the remaining ones were from the nearby states. Of the 325 children, only 58 (17.85%) were fully immunized, 156 (48%) were partially immunized, and 111 (34.15%) were non-immunized. Fifty-two (89.66%) of the fully-immunized children had immunization cards with them compared to 50 (18.73%) children who were either partially or non-immunized. All the partially-immunized patients and 83.78% of the non-immunized patients received OPV during the pulse polio immunization campaign. The coverage of pulse polio immunization was 96%.

Table 1 shows the demographic profiles of the study children in relation to the immunization status. Of the 58 completely-immunized patients, 51 (87.93%) were male while 58 (37.2%) of the 156 partially-immunized patients were male (p<0.001). Of the 104 patients from Delhi, 39 (37.5%) were completely immunized, and 19 (8.6%) of the 221 patients from outside Delhi were completely immunized. This difference was significant (p<0.001). Of Hindus (n=35), 37% received complete immunization, and 3.37% of Muslims (n=6) received complete immunization. Fathers of the non-immunized or partially-immunized cases were educated only up to primary level or less (82.77%, n=221) while 89.66% of fathers of the completely-immunized children were educated up to more than primary level. This was statistically significant (p=0.021). The effect of maternal education was also statistically significant (p=0.016). There was, however, no effect of birth-order or family type (p>0.05).

**Table 1.** Demographic profiles of patients studied

Variable	Completely immunized (n=58)		Partially immunized (n=156)		Non-immunized (n=111)		Total (100%)	p value
	No.	%	No.	%	No.	%		
Sex								
Male	51	34.46	58	39.19	39	26.35	148	<0.001
Female	7	3.95	98	55.37	72	40.68	177	
Place								
Delhi	39	37.50	39	37.50	26	25.00	104	<0.001
Outside	19	8.55	117	52.94	85	38.46	221	
Religion								
Hindu	52	35.37	63	42.86	32	21.77	147	<0.001
Other	6	3.37	93	52.25	79	44.38	178	
Family								
Joint	39	18.06	105	48.61	72	33.33	216	0.926
Nuclear	19	17.43	51	46.79	39	35.78	109	
Delivery								
Vaginal	6	3.00	103	51.50	91	45.50	200	<0.001
Caesarean	52	41.60	53	42.40	20	16.00	125	
Place of delivery								
Home	6	3.31	90	49.72	85	46.96	181	<0.001
Government centre	38	42.22	52	57.78	0	0	90	<0.001
Private	14	25.93	14	25.93	26	48.15	54	
Delivered by								
Doctor	58	42.03	60	43.48	20	14.49	138	
Trained *dai*	0	0	6	23.08	20	76.92	26	<0.001
Untrained	0	0	90	55.90	71	44.10	161	
Birth-order								
≤2	52	29.55	72	40.91	52	29.55	176	0.821
>2	6	4.03	84	56.38	59	39.60	149	
Education of fathers								
≤primary	6	2.64	130	57.27	91	40.09	227	0.021
>primary	52	53.06	26	26.53	20	20.41	98	
Education of mothers								
≤primary	12	5.15	117	50.21	104	44.64	233	0.016
>primary	46	50.00	39	42.39	7	7.61	92	

When the characteristics of the group with complete immunization were compared with the combined non-immunized or partially-immunized group, there was significant effect (p<0.001) of sex, education of fathers and mothers, place of delivery, and religion. Similar results were obtained when the group with complete or partial immunization combined was compared with the non-immunized group. When logistic regression was applied to compare the group having complete or partial immunization with the group having no immunization, the immunization status of children was affected by place of delivery [home vs hospital or private odds ratio (OR)=2.307, 95% CI-1.3–4.1], education of mothers (primary vs more than primary OR=6.94, 95% CI 3.016–15.099), and religion (Hindu vs rest OR=1.75, 95% CI 1.012–3.034) (data not shown).

The common reasons for partial immunization and non-immunization were: lack of knowledge about immunization (30.3%); immunization has side-effects (28.8%); lack of knowledge about subsequent doses (22.09%); lack of faith in the effectiveness of immunization (21.7%); and OPV was thought to be the only vaccination (20.9%) Table 2 shows the reasons for partial immunization or non-immunization. The single most common reason for partial vaccination was lack of knowledge about subsequent doses, and for non-immunization, the commonest reason was lack of knowledge about immunization. Other reasons were: doctors did not give advice (n=25); vaccine causes sterility (n=17); vaccine was not available (n=6); vaccinator was not available (n=6); and parents went to native places (n=2).

**Table 2.** Most frequent reasons for partial immunization/non-immunization

Reason	Partially immunized (n=156)		Non-immunized (n=111)		Total* (n=267)	
	No.	%	No.	%	No.	%
Lack of knowledge of immunization	7	4.5	74	66.6	81	30.3
Immunization has side-effects	40	25.6	37	33.3	77	28.8
Lack of knowledge of subsequent immunization	59	37.8	0	0	59	22.1
Lack of faith in effectiveness	23	14.7	35	31.5	58	21.7
OPV is the only vaccine	17	10.9	39	35.1	56	20.9
No vaccine for diarrhoea/ARI	28	17.9	6	5.4	34	12.7
Child is sick at scheduled visit	34	21.7	0	0	34	12.7
Reaction during first dose	32	20.5	0	0	32	11.9

*Some had multiple reasons;

ARI=Acute respiratory infection;

OPV=Oral polio vaccine

## DISCUSSION

Immunization is the most cost-effective intervention in child health. There is an impending risk of outbreak of vaccine-preventable diseases due to increasing urbanization, migration, increasing slums, high density of population, continuous influx of a new pool of infective agents, and poor coverage of primary immunization. Attempts to improve the coverage have been going on for years. The results of our study showed that only 58 (17.8%) children were immunized till one year of age, 48% were partially immunized, and 34.15% were non-immunized. Contrary to the results of previous studies on immunization which were conducted on outpatient children, the present study was carried out on admitted patients. A similar observation was reported by Mathew *et al.* who found that 25% of children were fully immunized (9) and Saxena *et al.* found that 30% were completely immunized (5). The higher coverage of immunization varying from 50% to 70% was observed in other studies (6–8, 11–13). Results of our study indicate a poor immunization status compared to the national average according to the NFHS 3 (2005–2006) which showed that 43.8% of children were fully immunized (4). Most previous studies included children from slums or rural districts, or were carried out among children attending the outpatient department. In this study, patients admitted to the paediatric ward were only included. This could be the reason for the lower coverage as non-immunized children are more likely to get infections and require admission in the paediatric ward. They have an increased level of morbidity and mortality. In the study in Delhi, the rate of immunized children was 71.7%, partially immunized 19.8%, and non-immunized 8.5% (7)**.** The immunization cards were available with 72.5% of them (7). Kar *et al.* found complete immunization in 69.3% of children in a slum area of Delhi (11). In our study, the coverage of complete immunization was 17.84%. This may be because of the fact that most of our study children were from slum areas of the nearby states where the immunization coverage is lower than Delhi.

The immunization cards were available with only 31.38% of the patients in this study compared to 74.4% in a study by Saxena *et al.* (5). The immunization cards were found in a higher percentage of the completely-immunized children compared to the partially-immunized and non-immunized children. This highlights the need for emphasizing the importance of record-keeping during immunization visits. All the partially-immunized and 83.78% of the non-immunized patients received OPV during the pulse polio immunization campaign. Many of them thought that pulse polio was the only immunization to be given or that the health workers would come to their home and immunize them. These issues need to be addressed to increase the coverage of immunization.

In our study, the fully-immunized children were predominantly male. The female children were less likely to receive complete immunization and more likely to remain in the non-immunized or partially-immunized group. These findings were also supported by the findings of other studies (5, 7, 13). Of the children (n=104) from Delhi, 37.5% were completely immunized compared to 8.55% of the completely-immunized children in those from outside Delhi, signifying a better immunization coverage in Delhi. We observed a low coverage of complete immunization among Muslim patients as has been found by other authors (8, 13).

Deliveries in the hospital, including those born by caesarean section, were more likely to be completely immunized (p<0.001). This may be because vaccination was started at birth, and parents were educated regarding subsequent vaccinations. Therefore, institutional deliveries should be promoted to increase the coverage of immunization. Education of fathers and mothers was also related to the low coverage of immunization in our study. Such findings were also observed by others (10, 11, 13). On logistic regression, three most common demographic factors affecting the immunization status were maternal education, religion, and place of delivery; hence, there is a need for maternal education.

The common reasons for partial immunization and non-immunization were: lack of knowledge about vaccination (30.3%); vaccination has side-effects (28.8%); lack of knowledge about subsequent doses (22.1%); lack of faith in the effectiveness of immunization (21.7%); OPV was thought to be the only vaccination (20.9%); vaccine should not be given if the child is suffering from minor illnesses, such as mild diarrhoea with no dehydration or acute respiratory infections (12.7%); child was sick on the scheduled date (12.7%); and minor reactions during previous vaccination (11.9%). Similar reasons have been reported in a study among urban slums of Lucknow district (8).

The single most common reason for partial vaccination was lack of knowledge about subsequent doses (22.09%); this highlights the need for training of medical officers and health workers about effective communication after vaccination regarding possible side-effects, their treatment, and the schedule for the next visit. The fact that minor illnesses, such as cough and diarrhoea, are not a contra-indication to vaccination needs to be told to the parents. Recently, it has been emphasized that satisfaction of clients, in terms of behaviour of health workers and information given by them, and easy accessibility are factors significantly different in completely-immunized and partially-immunized group (14). For non-immunization, the commonest reason was the lack of knowledge about vaccination.

The present study highlights that the immunization status of children admitted to atertiary-care hospital is low—reasons being low educational status of parents, lack of awareness, ineffective communication by healthcare providers, and misconceptions associated with immunization. These issues need to be addressed at the tertiary level to improve the coverage of immunization.

## References

[1] Frenkel LD, Nielsen K (2003). Immunization issues for the 21st century. Ann Allergy Asthma Immunol.

[2] Park K (2005). Principles of epidemiology and epidemiologic methods. Text book of preventive and social medicine.

[3] India (2000). Ministry of Health and Family Welfare. Department of Family Welfare. National population policy 2000.

[4] International Institute for Population Sciences (2007). National family health survey-3 (2005–06).

[5] Saxena P, Prakash D, Saxena V, Kansal S (2008). Assessment of routine immunization in urban slum of Agra district. Indian J Prev Soc Med.

[6] Manjunath U, Pareek RP (2003). Maternal knowledge and perception about the routine immunization programme—a study in semiurban area in Rajasthan. *Indian J Med*. Sci.

[7] Khokhar A, Chitkara A, Talwar R, Sachdev TR, Rasania SK (2005). A study of reasons for partial immunization and non-immunization among children aged 12–23 months from an urban community of Delhi. Indian J Prev Soc Med.

[8] Nath B, Singh JV, Awasthi S, Bhushan V, Kumar V, Singh SK (2007). A study on determinants of immunization coverage among 12–23 months old children in urban slums of Lucknow district, India. Indian J Med Sci.

[9] Mathew JL, Babber H, Yadav S (2002). Reasons for non-immunization of children in an urban, low income group in North India. Trop Doct.

[10] Bhatia V, Swami HM, Rai SR, Gulati S, Verma A, Parashar A (2004). Immunization status in children. Indian J Pediatr.

[11] Kar M, Reddaiah VP, Kant S (2001). Primary immunization status of children in slums areas of South Delhi: the challenge of reaching urban poor. Indian J Commun Med.

[12] Singh P, Yadav RJ (2000). Immunization status of children of India. Indian Pediatr.

[13] Yadav RJ, Singh P (2004). Immunization status of children and mothers in the state of Madhya Pradesh. Indian J Commun Med.

[14] Nath B, Singh JV, Awasthi S, Bhushan V, Singh SK, Kumar V (2009). Client satisfaction with immunization services in urban slums of Lucknow district. Indian J Pediatr.

